# Colorectal anastomotic leakage after conversion surgery for advanced endometrial cancer treated with lenvatinib plus pembrolizumab: a case report

**DOI:** 10.1007/s13691-024-00739-6

**Published:** 2024-12-10

**Authors:** Akitoshi Yamamura, Junzo Hamanishi, Koji Yamanoi, Masumi Sunada, Mana Taki, Rin Mizuno, Yukiko Okada, Ryusuke Murakami, Yuki Aisu, Hisatsugu Maekawa, Ken Yamaguchi, Masaki Mandai

**Affiliations:** 1 Department of Gynecology and Obstetrics, Kyoto University Graduate School of Medicine, 54 Kawaharacho, Shogoin, Sakyo-ku, Kyoto, 606-8507 Japan; 2 Department of Surgery, Kyoto University Graduate School of Medicine, Kyoto, Japan

**Keywords:** Endometrial carcinoma, Lenvatinib, Pembrolizumab, Anastomotic leakage, Conversion surgery

## Abstract

The combination therapy of lenvatinib plus pembrolizumab (LP) is increasingly recognized as an important second-line regimen for advanced or recurrent endometrial cancer (EC). However, the safety and efficacy of conversion surgery with low anterior rectal resection for unresectable EC following LP therapy is unknown. A 37-year-old woman was referred with unresectable EC with pleural fluid, peritoneal dissemination, and ascites. After the failure of first-line platinum-based chemotherapy, she was administered LP as second-line treatment. After 10 treatment cycles, uterine and peritoneal tumors significantly reduced in size, except the left ovarian metastatic tumor which became slightly larger. Cytoreductive surgery, including low anterior resection of the rectum and colorectal anastomosis, achieved complete resection. However, on postoperative day 11, the patient experienced an anastomotic leakage around the colorectal anastomosis site, necessitating a double-barreled colostomy and percutaneous drainage. She was discharged 15 days after the second surgery and resumed LP therapy after 44 days following the second surgery. We report a case in which conversion surgery after LP therapy was conducted for unresectable advanced endometrial cancer. Our findings indicate that if bowel resection is required, a longer preoperative withdrawal period may be necessary to prevent postoperative anastomotic leakage.

## Introduction

Endometrial cancer has an increasing incidence and prevalence rate and is the most common gynecological malignancy in high-income countries [1]. While platinum-based chemotherapy is typically the first-line treatment for advanced or recurrent endometrial cancer, most patients eventually develop resistance; thus, the limiting second-line treatment options [2]. The study 309/ KEYNOTE-775 trial demonstrated that combination therapy of lenvatinib and pembrolizumab (LP) significantly improved overall survival (OS) and progression-free survival (PFS) rates compared to second-line chemotherapies in patients who had been previously treated with platinum-based drugs [3]. Although the use of LP therapy was approved in the United States and Japan since 2021, the complete response rate for LP treatment is only 6.6% and the median PFS is 7.2 months. This highlights the need for identifying other better treatment strategies [4].

Conversion surgery, which aims for the complete resection of initially unresectable tumors after chemotherapy and/or immunotherapy, is an emerging concept in solid tumor treatment (Table 1) [5–7]. However, there are a lack of reports on the safety of conversion surgery with lower anterior rectal resection after LP therapy for advanced or recurrent endometrial cancer. This case report describes a patient with advanced endometrial cancer who underwent conversion surgery after LP therapy and subsequently had complications of rectal anastomotic leakage, leading to a reassessment of the indications for rectal resection post-LP therapy.

**Table 1:** Conversion surgery after lenvatinib with or without anti-PD-1 antibody therapy for solid tumors

Authors	Cancer type	Pretreatment	Surgical procedure	Surgical outcome	Withdrawal period of lenvatinib	Post-operative Complications
Yamazaki et al. (2023)^5^	Thyroid cancer	Lenvatinib	Total thyroidectomy, lateral neck node dissection	R0	7 days	None
Chen Z et al. (2024)^6^	hepatocellular carcinoma	Lenvatinib + Camrelizumab	Partial hepatectomy	R0	7 days	Mild bile leak
Hara et al. (2024)^7^	Renal cell cancer	Lenvatinib + Pembrolizumab	Lateral nephrectomy cavotomy, thrombectomy	R0	7 days	No information
This report	Endometrial cancer	Lenvatinib + Pembrolizumab	TAH, BSO, pOM, PS, LAR	R0	12 days	Colorectal anastomotic leakage

*TAH* total abdominal hysterectomy; *BSO* bilateral salpingo-oophorectomy; *pOM* partial omentectomy; *PS* peritoneal stripping of Douglas pouch; *LAR* low anterior resection of rectum; *R0* Complete resection of the tumor.a

## Case report

**Fig. 1:**
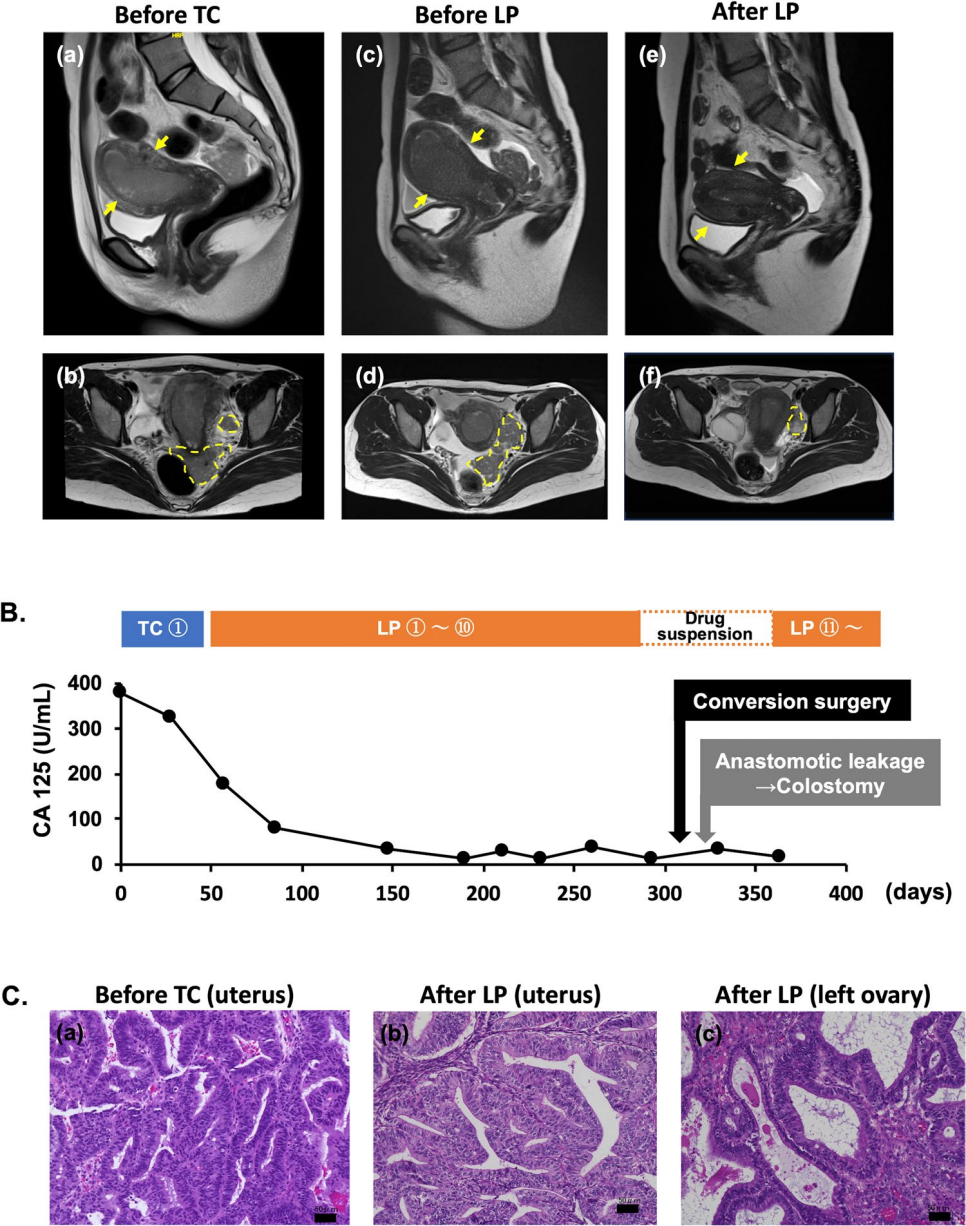
Magnetic resonance imaging (MRI), tumor markers, and histological findings during the clinical course of treatment. **A**. MRI during clinical course of treatment.**a**, **b** Images at diagnosis before paclitaxel and carboplatin (TC). Uterine tumor filled in uterine cavity (arrow), and peritoneal dissemination in Douglas pouch and left side of the pelvic floor (dotted line). **c**, **d** Images before lenvatinib and pembrolizumab (LP). Both uterine tumor (arrow) and the disseminated lesions (dotted line) were enlarged. **e**, **f** Images after LP. Both uterine tumor (arrow) and the disseminated lesions had shrunk, however the left ovarian tumor (dotted line) was enlarged. **B**. Time course of serum CA-125 during medication and surgery. After the initiation of the LP therapy tumor markers significantly decreased and later plateaued. **C**. Pathological images of primary uterine tumor and ovarian metastasis (hematoxylin-eosin staining). **a** Endometrial aspiration biopsy before TC. Well-differentiated endometrioid carcinoma with cribriform proliferation of the glands and scant solid areas was observed. **b** Uterine tumor tissue of conversion surgery. No significant change was observed compared to the aspiration biopsy specimen. **c** Left ovarian tumor tissue of conversion surgery. No significant difference between uterine tumor and aspiration biopsy specimen was observed. Original magnification of all pictures was high-power field (200x). Black bars mean 50 µm

A 37-year-old woman presented with abnormal bleeding and cough. The ultrasonography revealed pleural fluid retention, ascites, and large pelvic tumors. Magnetic resonance imaging (MRI) and contrast-enhanced computed tomography (CT) confirmed endometrial tumor with multiple dissemination in the Douglas pouch, right pleural effusion, and ascites (Fig. 1A). Positron emission tomography-CT showed no lymph node or distant metastasis. Cytological smear and cell block of pleural effusion were negative. Serum cancer antigen (CA)125 and CA19-9 levels was 379 U/mL and 230 U/mL, respectively, before chemotherapy (Fig 1B). Endometrial biopsy indicated grade 1 endometrioid carcinoma (Fig. 1C) and immunohistochemistry findings indicated proficient mismatch repair (MSH6 and PMS2) and wild type p53. Pleural effusion was nonmalignant and peritoneal dissemination was confined to the pelvic cavity; therefore, the patient was diagnosed with unresectable stage IIIB endometrial cancer and started on systemic chemotherapy with paclitaxel and carboplatin (TC).

After one course of TC, the patient’s abdominal distension worsened. Further, the MRI showed that both the intrauterine lesion and peritoneal masses had enlarged and a new left ovarian tumor was observed (Fig 1A). LP therapy was selected as a second-line treatment, leading to tumor shrinkage and a significant decrease in tumor markers (Fig. 1B). Lenvatinib administration was initiated at 20 mg/days and was gradually reduced to 4 mg/days due to systemic edema and proteinuria. Four months after starting LP therapy, the pleural effusion resolved. By 6 months, peritoneal dissemination was not visible on MRI; however, the primary lesion remained and the left ovarian metastasis slightly increased (Fig. 1A). Given the possibility of resistance to LP therapy in the future, conversion surgery was planned after 10 courses of LP therapy. To avoid perioperative complications, the administration of pembrolizumab was stopped for 4 weeks and that of lenvatinib was stopped for12 days before surgery.

**Fig. 2:**
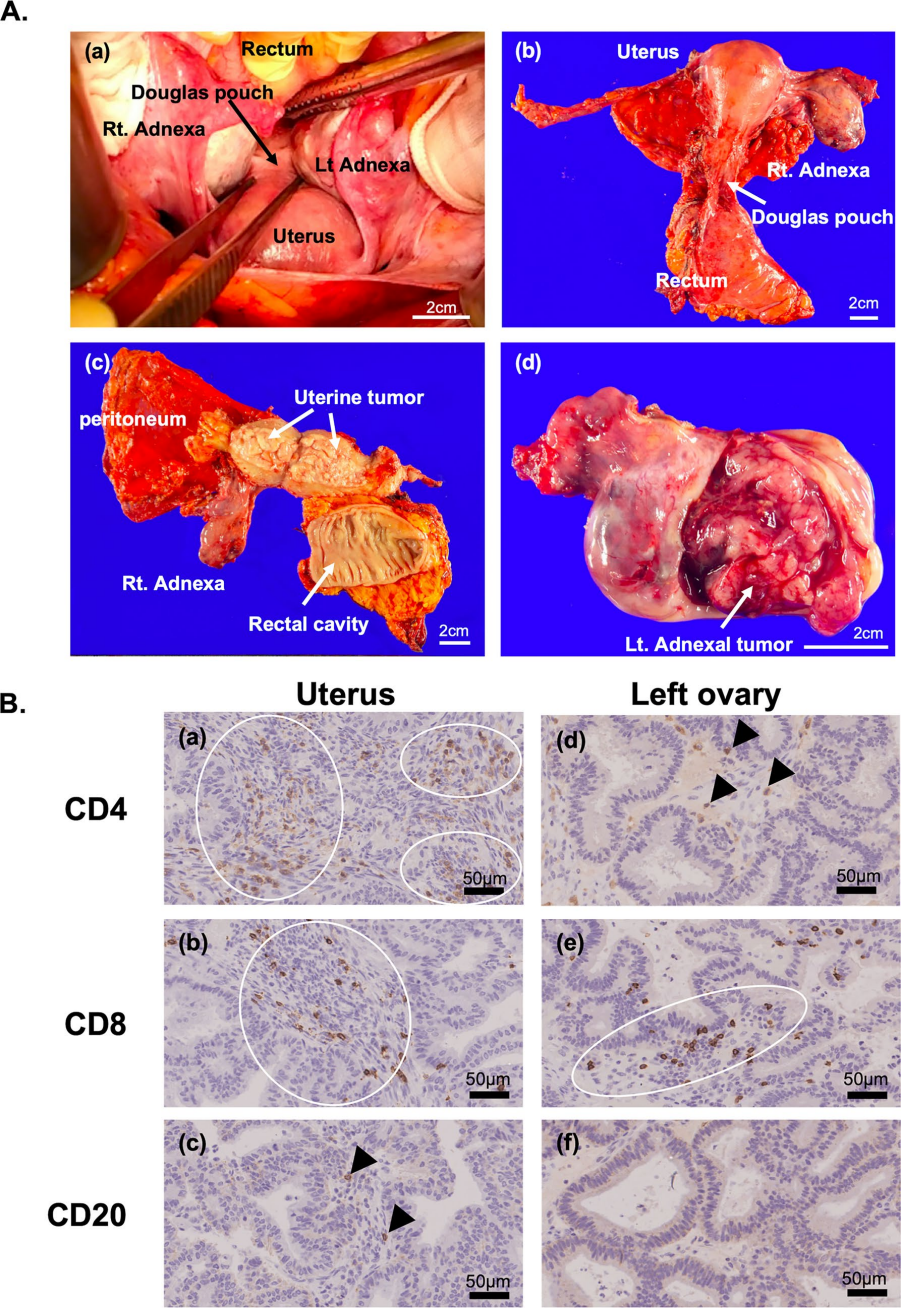
Intraoperative, macroscopic, and pathological findings. **A**. Intraoperative finding and specimen images. **a** Intraoperative finding. Miliary dissemination in the Douglas pouch was found but no other macroscopic peritoneal tumor was detected. The left ovary was enlarged to 5 cm. **b** Specimens of uterus with pelvic peritoneum and rectum and right adnexa. Miliary dissemination in Douglas pouch was found. **c** Specimen of the incised uterus and rectum. The uterine cavity was filled with papillary tumor. **d** Specimen of the incised left adnexa. The left ovary was enlarged and was filled with papillary tumor. Black bars mean 2 cm. **B**. Representative immunohistochemical findings (IHC) for lymphocyte surface antigen in uterine tumor and left ovarian metastatic tumor. **a** CD4-positive cells in uterine tumor. White ring showed many positive cells. **b** CD8-positive cells in uterine tumor. White ring showed many positive cells. **c** CD20-positive cells in uterine tumor. Arrowheads showed positive cells. **d** CD4-positive cells in left ovary. Arrowheads showed positive cells. **e** CD8-positive cells in left ovary. White ring showed many positive cells. **f** CD20-positive cells in left ovary. No positive cell was found. Original magnification of all pictures was high-power field (200x). Black bars mean 50 µm

Intraoperatively, the left ovary was enlarged; however, peritoneal dissemination in the Douglas pouch was reduced to miliary grain size, with no other peritoneal lesions (Fig 2A). Total abdominal hysterectomy, bilateral salpingo-oophorectomy, peritoneal stripping, partial omentectomy, and low anterior resection of the rectum with an end-to-end anastomosis stapler were performed. Ascitic fluid cytology was negative and complete resection was achieved without complications. The surgery duration was 8 h 13 min with a blood loss of 1260 mL. Postoperative pathology revealed that the uterine tumor was grade 1 endometrioid carcinoma, and the left ovarian tumor was deemed metastatic from the uterine primary. No rectal infiltration was noted (Fig. 2A). Immunohistochemistry showed a locally higher infiltration of CD4-positive and CD8-positive cells in the uterine tumor compared to the ovarian tumor and scarce presence of CD20-positive cells in both tumors (Fig 2B).

**Fig. 3:**
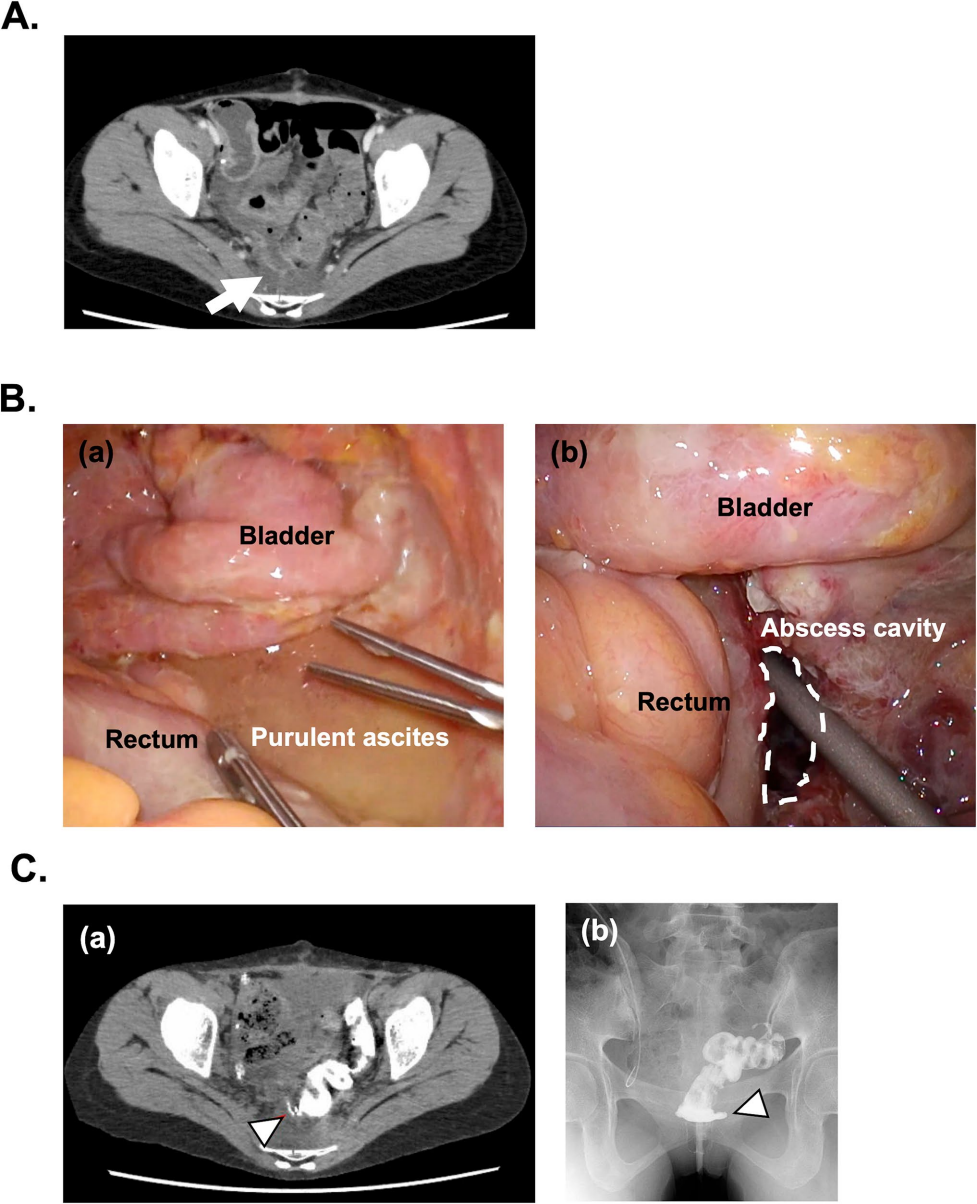
Rectal anastomotic leakage and second operation. **A**. Computed tomography on postoperative day 11. Intraabdominal free air (arrowhead) and pelvic abscess around rectal anastomosis (arrow) were found. **B**. Intraperitoneal findings by exploratory laparoscopy. **a** Purulent ascites was observed around the anastomotic site. **b** Pelvic abscess was removed and a drain was inserted into the abscess cavity (dotted area). **C**. Postoperative contrast enema for detecting rectal anastomotic leakage on second-postoperative day 8. **a** CT scan following gastrografin contrast enema revealed the back side of leakage around rectal anastomosis (arrowhead). **b** Gastrografin contrast enema also indicated rectal anastomotic leakage (arrowhead)

The postoperative course was uneventful. The patient passed gas on postoperative day (POD) 1, started oral intake on POD 3, and had a bowel movement on POD 6. The blood test showed no abnormal findings on POD 7; white blood cell (WBC) count was 5000/µL and the C-reactive protein (CRP) level was 0.4 mg/dL. However, the patient developed lower abdominal pain and peritoneal irritation on POD 10, and the WBC count increased to 28,000/μL and CRP level increased to 5 mg/dL on POD 11. CT scan revealed an unusually large amount of free air and a suspected abscess at the rectal anastomosis site (Fig. 3A). Emergent exploratory laparoscopy demonstrated a perforation of the peritoneal abscess around the rectal anastomosis (Fig. 3B). After washing the abdominal cavity, a double-barreled colostomy was created in the transverse colon with abscess drainage in the Douglas pouch. Contrast enema and plain pelvic CT scan following gastrografin contrast enema revealed posterior rectal anastomotic leakage on second-POD 8 (Fig. 3C). Postoperative recovery was uneventful with good infection control and the patient was discharged on second-POD 15. LP therapy was resumed after second-POD 44 and did not have any complications or recurrence for 3 months.

## Discussion

This case demonstrated a drawback of conversion surgery for rectal resection after LP. We also showed the potential effectiveness of LP therapy promoting the transformation of unresectable advanced, chemo-resistant endometrial cancer to resectable cancer.

Rectal anastomotic leakage is a fatal complication in colorectal surgery. Previous reports of colon cancer have reported an incidence rate of rectal anastomotic leakage of 3.2–7.3% in colorectal cancer and 2.9–4.2 % in ovarian cancer [8–10]. The known risk factors for colorectal anastomotic leakage after colorectal surgery are low anastomotic levels, malnutrition, anemia, liver comorbidity, additional surgery, and long operation time [8–10]. However, the pre-operative administration of TC therapy is not considered a risk factor for anastomotic leakage [9]. In the present case, a long operation time (over 8 h) and additional surgeries (such as hysterectomy and pelvic peritoneal stripping) might increase the risk of anastomotic leakage. However, the patient had normal nutritional status, good liver function, and no severe complications during the operation. Besides, the phase 3 trial of KEYNOTE-775, which compared treatment with LP therapy versus conventional chemotherapy in patients with advanced or recurrent endometrial cancer, information on the risks of bowel resection after LP therapy was not reported. Although LP therapy for advanced endometrial cancer and renal cell carcinoma was approved by Food and Drug Administration since 2021, there is only a case report of conversion surgery without bowel resection for renal cell carcinoma before LP therapy (Table 1) [7]. We have described three cases of conversion surgery with complete tumor resection after lenvatinib with or without immune checkpoint inhibitors for thyroid, hepatocellular, and renal cancers. Although all these patients had a 7-day-withdrawal before conversion surgery, mild bile leak was reported in only one case of partial hepatic resection after lenvatinib and the anti-PD-1 antibody camrelizumab for hepatocellular carcinoma (Table 1). However, none of the patients underwent gastrointestinal or colorectal resection. In our case, with lenvatinib and pembrolizumab, a longer preoperative lenvatinib withdrawal period (12 days) was used; however, postoperative colorectal anastomotic leakage occurred (Table 1).

Lenvatinib is an oral multi-kinase inhibitor that selectively inhibits vascular endothelial growth factor (VEGF) receptors 1–3, fibroblast growth factor (FGF) receptors 1–4, and other proangiogenic tyrosine kinases and has a potent antiangiogenic property [11]. The inhibition of VEGF receptors, through their antiangiogenic properties, reduces the capillary bed in tissues by 22–59% [12]. FGF signaling plays a critical role in wound healing by promoting processes such as cell proliferation, angiogenesis, and tissue remodeling. FGF signaling stimulates the migration of fibroblasts to the wound site, enhances collagen production, and supports the formation of new blood vessels, all of which are essential for effective tissue repair and regeneration [13]. Therefore, the surgical decisions after administration of antiangiogenic drugs like lenvatinib must be taken with caution because of the ability of the drug to delay wound healing [14]. However, the surgery of the low anterior resection of the rectum after lenvatinib therapy has not been reported, though some reports have demonstrated that lenvatinib increased the risk of gastrointestinal perforation [3, 14, 15]. The half-life of lenvatinib is approximately 28 h [11]. In surgeries for other types of cancer after lenvatinib treatment, presurgical pauses in medication ranging 4–10 days have been reported [5, 6, 16]. Based on these considerations, we performed conversion surgery after a 12-day suspension of lenvatinib administration. However, an anastomotic leakage was diagnosed on POD 11. Anastomotic leakage typically occurs within the first 7 days, postoperatively [17]. In this case, no signs of infection (which are common signs to anastomotic leakage) were observed and the leakage occurred later than the typical timeframe after colorectal surgery. The antiangiogenic effects of lenvatinib may have resulted in reduced blood flow to the anastomotic site, thereby delaying the healing process. Although generalizing from a single case is not possible, these findings suggest the need for greater caution in intestinal surgery after lenvatinib administration. Moreover, bevacizumab which is an antiangiogenic antibody, is one of the risk factors for anastomotic leakage [18]. The appropriate interval between the last dose of bevacizumab and elective surgery is unknown; however, the half-life of bevacizumab is estimated to be 20 days. Thus, the National Comprehensive Cancer Network guidelines suggest withholding bevacizumab at least 6 weeks before surgery [18]. Owing to the VEGF inhibitory effect, the capillary density in normal tissues may remain reduced even after lenvatinib discontinuation and the subsequent decline in blood concentration, potentially disrupting normal tissue architecture. Currently, the evidence is limited regarding the appropriate preoperative discontinuation period for lenvatinib and the time needed for the tissues to recover to a safe state for surgery remains unclear. However, considering the antiangiogenic mechanism of lenvatinib, a preoperative withdrawal period close to that recommended for bevacizumab may be advisable.

Pembrolizumab is one of the immune checkpoint inhibitors and has the potential to induce immune-related adverse events of bowel diseases such as colitis and intestinal perforation [19]. However, a meta-analysis investigating the effects of a combination of immune checkpoint inhibitors with neoadjuvant chemotherapy for the preoperative treatment of locally advanced esophageal cancer demonstrated that the immune checkpoint inhibitors did not increase the incidence of postoperative anastomotic leakage [20]. Additionally, in a phase 1 study of neoadjuvant nivolumab monotherapy for resectable gastric cancer, the incidence of anastomotic leakage reported in 2 out of 30 patients, and the frequency was not higher than that after neoadjuvant chemotherapy with conventional chemotherapeutic agents [21]. As indicated by these studies, these reports suggest that immune checkpoint inhibitors are not directly associated with an increase in anastomotic leakage. In this case, pembrolizumab was discontinued 1 month before conversion surgery, in accordance with the protocol reported in previous studies [20, 21]. Therefore, we believe that the preoperative administration of pembrolizumab is unlikely to be a risk factor for anastomotic leakage in this patient.

Regarding the diagnosis of the left ovarian tumor, based on the Scully’s criteria, the possibility of a double primary cancer cannot be entirely excluded [22]. However, in this case, endometriosis was not observed around the ovarian lesions. We have previously investigated synchronous primary corpus and ovarian cancers and reported that endometriosis is a significant characteristic of synchronous primary cancers [23]. Considering this along with the overall clinical picture, we concluded that this represents ovarian metastasis of endometrial cancer.

Antitumor efficacy of conversion surgery after LP therapy for unresectable uterine cancer is still unknown. In the KEYNOTE-775 trial, the median PFS for LP therapy in the proficient mismatch repair population was reported to be 6.6 months [3]. In this case, the patient mostly responded to LP therapy at 6 months; however, a slight increase was noted in tumor mass in some areas suggesting imminent LP therapy resistance. Although the KEYNOTE-775 trial did not include patients in which conversion surgery was performed after LP, we established that surgical treatment at this timing should be appropriate in our case. Pathological examination of the resected specimen showed that a significant number of viable tumor cells were intact, suggesting the limited efficacy of LP (Fig 1C). The left ovarian tumor, which had increased in size, exhibited fewer tumor-infiltrating CD4-positive and CD8-positive cells compared to the uterine tumor, suggesting different therapeutic responses between the two lesions (Fig. 2B). The lower presence of both CD4-positive and CD8-positive T cells in the growing left ovarian tumor than in the uterine tumor suggests that immune tolerance after LP therapy may have occurred, potentially leading to resistance to LP therapy in the left ovarian tumor.

At present, there is no evidence to support neoadjuvant setting with LP as a precursor to conversion surgery in advanced endometrial cancer. If more cases where surgery become possible after LP and has favorable outcomes are observed and reported, clinical trials using LP therapy prior to surgery in such cases could be explored.

In conclusion, we conducted a conversion surgery after LP therapy for unresectable advanced endometrial cancer. However, postoperative vigilance for anastomotic leakage is warranted.

## Data Availability

My manuscript has no associated data.
